# Risk factors associated with self-medication among women in Iran

**DOI:** 10.1186/s12889-019-7302-3

**Published:** 2019-08-01

**Authors:** Mahmood Karimy, Majed Rezaee-Momtaz, Mahmoud Tavousi, Ali Montazeri, Marzieh Araban

**Affiliations:** 1 Social Determinants of Health Research Center, Saveh University of Medical Sciences, Saveh, Iran; 2 Department of Health Education and Promotion, Public Health School, Ahvaz Jundishapur University of Medical Sciences, Ahvaz, Iran; 3 Health Metrics Research Center, Iranian Institute for Health Sciences Research, ACECR, Tehran, Iran; 4 Social Determinants of Health Research Center, Ahvaz Jundishapur University of Medical Sciences, Ahvaz, Iran

**Keywords:** Theory of planned behavior, Self-medication, Women

## Abstract

**Background:**

Self-medication is a public health concern that may create several problems such as increase in drug resistance, raise in drug use per capita, and creating side effects. This study was conducted to determine predictors of self-medication based on the theory of planned behavior (TPB) among the Iranian women.

**Methods:**

This was a cross sectional study. A sample of married women completed a validated, anonymous, self-administered questionnaire. The questionnaire included items on demographic variables, knowledge, and TPB structures, and the checklist of women’s self-medication practices. The study was conducted in six urban health centers of Shush and Zarandieh in Iran during January to July 2016. Data were analyzed using SPSS 23.0 applying t-test and logistic regression.

**Results:**

A total of 360 women took part in the study. The results showed that 76% of women had a history of self-medication and 98.9% stored drugs at home. The most important reasons for self-medication were perceived self-medication harmless (41%), having history of a disease (35.5%), and availability of medications at home (34%). The most frequent diseases for self-medication were fatigue, weakness, and anxiety (24%), and fever (20%). The results obtained form logistic regression analysis indicated that age, place of residence, education level, health insurance status, as well as all constructs of the TPB were significant predictors for self-medication.

**Conclusion:**

The findings indicated that the prevalence of self-medication among Iranian women was high. Since women have important role in shaping the family health, the issue of high prevalence among this population should be considered as a serious problem in Iran. In this regard, it is recommended to consider factors affecting self-medication among women to reduce this health threatening factors.

## Background

Self-medication (SM) is a practice in which people use drugs to improve their health that might be different from the help or recommendation of health experts [1]. SM has different forms including taking one or more medications without physicians’ prescription, using the previous drug in similar situations, using drugs available at home and not adhering to the physician’s recommendation [2–4].

SM is a critical health concern [4] that might cause several problems such as antibiotic-induced drug resistance, raised drug use per capita, non-desired treatment, and drug toxicity [5]. Studies have shown that SM is accounted for 3% of congenital anomalies. In addition, in some occasions SM could impose extra costs on health care system [4, 6].

SM has been accounted for 67% of the global burden of disease. It has been reported that the rate of SM among Iranian is much more than the global rate [6, 7]. Reports show that 399 drugs per person per year are taken by an Iranians, which is 2 to 4 times higher than the global use [7]. In the United State of America 42% of people take drugs without physician’s prescription [8]; this rate is reported to be 91% in Indonesia, [9]; and 57% in Indian women [10]. In Iran, the prevalence of SM is equal to 36 to 83% in different parts of the country [7]. As such it seems that factors effecting SM in different sub-groups of the population are different and of paramount importance. Women are considered to be an appropriate group for implementing health programs due to being in more contact with family members and with the health care system during pregnancy and the child-growth monitoring period. They also serve as a key role model for their children [6]. However, for some physiological reasons such as dysmenorrhea, premenstrual syndrome, and pregnancy complications they usually consume medication without consulting doctors [5, 11, 12]. Therefore, targeting women for educational interventions in this context might provide better outcomes [5].

Studies showed that a vast majority of people do not have proper knowledge and belief regarding the side effects of SM [7, 13]. SM might lead to the occurrence of adverse drug reaction, Wrong dosages, drug resistance, etc., in addition to, SM could play an important role in health costs rise [14–16]. Given the increasing access to a wide variety of medications in the community and the critical role of individuals in selecting and consuming medications in order to improve their own health, a number of investigators employed theories and models to identify factors that might affect and change people’s behaviors [4]. As such Theory of Planned Behavior (TPB) is commonly used to address the issue.

The TPB is a theory that was developed by Icek Ajzen and links beliefs and behaviors [17]. The theory states that attitude toward a behavior, subjective norms, and perceived behavioral control, together shape an individual’s behavioral intentions and behaviors. The theory has its root in outcome expectancy [18]. Based on the theory of outcome expectancy people involve in a given behavior when they perceive that a particular behavior can lead to a positive outcome or the effectiveness of the proposed preventive behavior in reducing the vulnerability to negative outcomes is guarantied. Several studies have shown the applicability of TPB in health education practices [17, 19]. Considering the lack of a theory based study regarding the SM and the fact that SM is a behavioral health problem, the current study was conducted to determine the risk factors associated with self-medication based on the theory of planned behavior.

## Method

### Design, procedure and the study sample

This was a descriptive study on a sample of married women covered by urban health centers of Shush (a city in south) and Zarandieh (a city in central region), Iran. The Shush has a population of 43534 inhabitants. The population of married women in Shush is estimated at 7968 inhabitants.. The Zarandieh has a population of 63907 inhabitants. The population of married women in Zarandieh is estimated at 10721 inhabitants. An expert panel including one epidemiologist, one maternal child specialist, and four health education and promotion specialists confirmed the selection of the cities. The study was conducted in six urban health centers of Shush and Zarandieh during January to July 2016.

The inclusion criteria were being married, having a child under 6 years old, and lack of any chronic or specific diseases like diabetes, hypertension, and cancer. The exclusion criterion, on the other hand, was rejction to participate in the study. Considering the SM prevalence of 86% in a previous study [7] and the estimate precision of 4% at confidence level of 95%, the number of samples required to participate in the study was considered to be 289. However, in practice 380 people were entered to the study in order to increase the study power. Proportinal to the population of two cities, 150 people from Shush (Khoozestan province) and 230 participants from Zarandieh (Markazi Province) were included in the study. Indeed first, the list of health care centers was provided. Four health care centers from Shush and six from Zarandieh were selected through simple random sampling method. Then, based on the number of women attending to the centers, study samples were selected via random sampling from each center. Finally after analyzing the received questionnaires and discarding the incomplete questionnaires, the final analysis was carried out on a total of 360 questionnaires (140 questionnaires from Shush and 220 questionnaires from Zarandieh).

### Measures

A self-designed questionnaire was used to collect the data. The questionnaire included 3 parts. The first part included 10 items on demographic variables. The second part consisted of items on the constructs of TPB (including 9 items for measuring knowledge about SM, 7 items for measuring attitude toward SM, 4 items for perceived behavioral control (PBC), and 4 items for subjective norms (SN) and a 10-items checklist for measuring SM over the last 3 months for some common diseases with the probability of self-medication). The third part of the questionnaire examined the reasons for SM with 10 statements. For example, we asked respondents why you used a drug without doctor’s prescription and they could choose the statement(s) that best described their reasons such as ‘ It was available at home or from others’ or ‘The disease was unimportant’. The content validity of the questionnaire was confirmed by a panel of health education and health promotion specialists and a number of physicians (content validity index = 1, content validity ratio = 1). Face validity was examined by a sample of 20 women and the results was promising (item impact score = 5). The reliability of the questionnaire was assessed by Cronbach’s alpha coefficient. A sample of 25 women who were similar to the study population in terms of demographic features completed the questionnaire and the alpha values were as follows: 0.79 for knowledge, 0.82 for attitude, 0.80 for PBC, and 0.86 for SN.

### Scoring

The items of attitude toward the behavior, abstract norms, and perceived behavioral control were designed based on a 7-point Likert scale varying from ‘strongly agree’ (1 point) to ‘strongly disagree’ (7 points). For the knowledge, the correct answer assigned 1 and the incorrect answer as 0. The scoring for the constructs of TPB ranged from 1 to 7 for each item. The items within the checklist (the practice of self-medication) and reasons for SM were set as yes/no format.

### Statistical analysis

All data analyses were conducted according to a pre-established analysis plan through SPSS 23 (SPSS, Inc., Chicago, IL, USA). Independent sample t-test was used to compare the mean scores of the constructs of the TPB between two groups (those with and without SM). Logistic regression was performed to determine the association between dependent variables and self-medication. Since the mother’s job, the number of children, age, husband’s age, and education, income were not significant in univariate analysis these were not included in the multiple logistic regressions models. The significance level was set at 0.05 levels. This approach has been reported earlier [20].

### Ethics

The Research Ethics Committee of the Saveh and Ahvaz Jundishapur University of Medical Sciences approved the study (Number: IR.SAVEHUMS.REC1396.15, IR.AJUMS.REC.1398.056.). All participants completed a written informed consent.

## Results

**Fig. 1:**
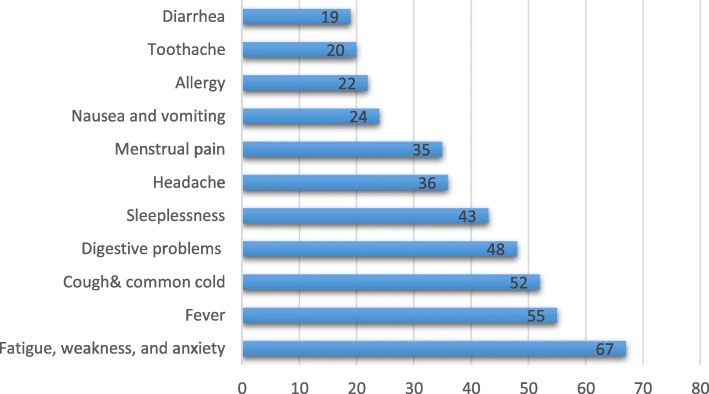
Conditions treated by self-medication practice as stated by women

In all 380 questionnaires were completed. Of these 20 questionnaires were excluded due to missing data and thus the final analysis was carried out on 360 questionnaires. The mean age of participants was 36.4 ± 6.2 years. Overall, 76% of the sample reported that they had a history of SM, Of these, 69% indicated that husband or a friend encouraged them to take drugs without prescription and almost all women (98.9%) reported that they store drugs at home; 75% had recommended a drug to friends and relatives over the last 3 months, 81% had drug prescription for first degree family members (children/spouse), and 80% believed that SM is the same as self-care. As indicated in Table 1, the most important reasons of SM were: perceived self-medication harmless (41%), having history of a disease (35.5%), and availability of medications at home (34%). In addition the highest frequency of self-medication by a disease was for fatigue, weakness, and anxiety (24%) and the lowest was for diarrhea (6.8%). Self-reported conditions treated by self-medication among women are presented in Fig. 1.

**Table 1:** The reasons of self-medication from the perspectives of women

Reasons for self-medication	Number	(%)
Considering medications harmless	146	41
Having history of the disease	128	35.5
Medications availability at home or from others	122	34
Easy and no-prescription delivery of medications from pharmacies	107	30
Considering the disease unimportant	106	29
Insistence of others	91	25
Expensive fees of medical appointments	50	14
Distrust of doctors	42	11.6
Having no access to doctors	33	9
Having not enough time for medical appointments	30	8

In univariate analysis the association between self-medication and the reasons for such practice was not significant but it was significant with knowledge, attitude, PBC, SN, age, place of residence, health insurance, and education level. However, the results obtained from multiple regression analysis indicated that some demographic variables such as age, place of residence, education level, and not having health insurance and all constructs of the TPB were significant predictors for self-medication. Among the TPB constructs the PBC, SN, attitude, and knowledge were the most important predictors (Table 2).

**Table 2:** Binary logistic regression analysis of reasons associated with self-medication

Behavioral constructs	Self-medication (*n* = 276)	Non-self-medication (*n* = 84)	OR (95% CI)	*P*
Variable				
Knowledge (mean, SD)	4.15 ± 1.80	7.63 ± 1.57	0.57 (0.35–0.94)	0.03
Attitude (mean, SD)	21.78 ± 4.25	27.41 ± 4.18	0.68 (0.51–0.92)	0.02
Perceived behavioral control (mean, SD)	9.68 ± 2.37	12.04 ± 2.11	0.74 (0.60–0.89)	0.001
Subjective norms (mean, SD)	11.97 ± 2.01	16.21 ± 2.15	0.72 (0.58–0.91)	0.008
Demographical factors	No. (%)	No. (%)		
Age				
≤ 19	6 (1.6)	11 (3)	1.0 (Ref.)	
20–29	58 (16.1)	27 (7.5)	1.56 (1.18–2.08)	0.004
30–39	111 (33.3)	28 (7.7)	1.94 (1.04–3.79)	0.041
40≥	101 (25.5)	18 (5)	2.21 (1.20–4.20)	0.01
Place of residence				
Urban	12 (3)	40 (11)	1.0 (Ref.)	
Rural	264 (73)	44 (12)	1.55 (1.15–2.1)	0.004
Health insurance				
Yes	250 (69)	73 (20)	1.0 (Ref.)	
NO	26 (7)	5 (1.3)	1.41 (1.20–1.87)	0.001
Education level				
Higher	94 (26)	35 (9.7)	1.0 (Ref.)	
Secondary	115 (32)	33 (9)	1.21 (1.09–1.35)	0.001
Primary	62 (17)	13 (3.6)	1.25 (1.06–1.27)	0.003
Illiterate	5 (1.3)	3 (0.8)	1.46 (1.18–1.91)	0.001

## Discussion

The results showed that a high proportion (76%) of the study sample in this study had a history of SM over the last 3 months. Similar findings were reported for women from Uganda [11], India [21], Chile [22] and Italy [23]. However, lower prevalence of self-medication was reported from other regions [24, 25]. For instance, the prevalence of self-medication was 51% in Slovenia [26], 55.3% in Pakistan [27], and 55% in Egypt [28]. SM may lead to problems such as increased per-capita drug use, drug resistance, non-optimal treatment, poisoning, and unwanted side effects [23, 27]. Moreover, SM in women is of more importance, since they experience sensitive periods in their life including pregnancy and lactation. They also serve as the role model for the family members. Hence, it seems necessary to take effective interventions to prevent and reduce SM as an acute health problem in women.

The high prevalence of SM in this study can be attributed to multiple factors. For instance, the easy access to drugs without prescriptions. Such an observation also was reported by similar previous investigations on SM [4, 29]. The study of Motola in Italy [30], Uehleke in Germany [31], and Bonner in the USA [29] also are consistent with the current work. The low perceived threat could be named as another factor for SM in our study since most people indicated that they use drugs without doctors’ prescriptions because they see drugs harmless or they considerthe disease unimportant. In line with the present study, in the study by Ahmad et al. in India [32], Zafar et al. in Pakistan [27], and Yu in China [33] the low perceived threat was identified among the major reasons of self-medication. The availability of drugs at home can be referred to as another cause for the high SM in our study, which is in line with previous studies [33–35]. The high prevalence of SM in this study can also be explained by existing social norms in Iran. The results showed that one-fourth of the study samplehad SM upon the persistence of others and 69% were encouraged by their husbands/friends for SM. Considering the adverse consequences of taking drugs stored at home by children, healthcare professionals should think about the risk of accidental intoxication among children. As such, providing adequate counseling to mothers about the potential hazards of drugs stored at home is strongly recommended.

Consistent with other studies [4, 18, 19], our results showed that perceived behavioral control had a more important role than the other constructs of the TPB in predicting for SM. It should be noted that the effectiveness of this construct in reducing high-risk behaviors is proven, so health experts must increase the individual’s PBC by improving his/her required skills and knowledge. Based on our findings, the attitude was a significant variable in predicting for SM, and that the non-SM scored higher on the attitude compared with the SM group. Here, 41% of the women believed that the drugs they used are harmless and over two-thirds of them considered SM some kind of self-care, which suggests the prevalence of wrong attitudes towards SM. In accord with our findings, in the study by Ocean [11], people with SM had lower scores for attitude. Also, the study of Panagakou [36] indicated that the low attitude was positively related to SM. Overall, it can be stated that it is beneficial to conduct campaigns to change false beliefs through mass media.

Studies on the relationship between knowledge and self-medication showed that wrong and inadequate information is a key contributing factor to high prevalence of SM. Consistent with other works [13, 37], our study showed that the knowledge level of the participants was significant predictor of their SM. In a study by Bajcetic & Jovanovic, parents with lower knowledge had more self-prescription of antibiotics for their children [38]. Elsewhere, Widayati et al. found that knowledge and attitude are the major factors of SM behavior [39].

The results of the present study showed that there was a significant association between age and self-medication where older age was associated with higher probability of SM. This finding is very important considering that from biological perspective liver and kidney are responsible for metabolism of drugs and these organs loss their optimal performance in old age [40] causing an increase in the occurrence of drugs’ side-effects (probably due to the prolonged exposure time) [4]. The prevalence of SM among different age groups is reported in most previous studies [13, 37, 41]. In addition, our results showed that living in rural areas increases the chance of SM as compared to those who were living in urban areas. Perhaps such observation might be attributed to lack of access to physicians and health services in rural areas, which in turn leads to storage of drugs at home and self-medication. It could also be explained that illiteracy or low literacy and consequently low knowledge of the side effects of self-medication are other reasons for increased self-medications by those who are living in rural areas. The higher SM prevalence among those who live in rural areas are reported in previous studies [11, 32]. The literature recommended interventions for enhancing knowledge about the side effects of SM through media such as magazines, radio, and TV to prevent SM among people that live in rural areas.

The results showed that the lack of medical insurance increases the chance of self-medication because people without medical insurance preferred to obtain drugs directly from pharmacies due to the high fees of medical appointments. This result might indicate a need for public insurance for all people in the community. Finally our study demonstrated that the education level is among the important factors affecting SM prevalence: as the education level decreases, the chance of SM increases. As previously evidenced [13, 42], this behavior could be explained by the low health literacy of the low-educated people and consequently their low knowledge of the risks of self-medication. Researchers believe that the role of education in health and health behaviors is more important than that of financial income [18].

### Limitation

Self-reported drug use, the possibility of memory bias (SM over the last 3 months) and non-participation of men in the study could be named as the “limitations” of the present study. The current study assessed behavioral determinants on self-medication based on TPB, which might not perfectly describe all factors associated with self-medication.

## Conclusion

The findings indicated a high prevalence of SM among Iranian women. Considering the important role of women in the health of family and society, this subject must be considered as a health threat in Iran and must be dealt with properly. In this regard, it is recommended to consider factors affecting SM among women to reduce this health threatening factors.

## Data Availability

Upon request, we can offer onsite access to external researchers to the data analyzed at Ahvaz Jundishapur University of Medical Sciences, Ahvaz, Iran. To do so, Dr. Araban should be contacted.

## Abbreviations

PBC: Perceived behavioral control
SM: Self-medication
SN: Subjective norms
TPB: Theory of planned behavior
